# Toward interface-adaptive silicon anodes: From 3D SEI to dynamic coupled interphases

**DOI:** 10.1016/j.xinn.2026.101360

**Published:** 2026-03-30

**Authors:** Hong Yin, Yingqi Cao, Yaru Wang, Junlin Huang, Sitaramanjaneya Mouli Thalluri, Zhipeng Yu

**Affiliations:** 1 Key Laboratory of Hunan Province for Advanced Carbon-Based Functional Materials, School of Chemistry and Chemical Engineering, Hunan Institute of Science and Technology, Yueyang 414006, China; 2 International Iberian Nanotechnology Laboratory (INL), 4715-330 Braga, Portugal

**Keywords:** solid-electrolyte interphase, adaptive interfaces, hierarchical chemical coupling, self-healing materials, silicon anodes

## Abstract

This review examines the development of silicon anodes in lithium-ion batteries, focusing on modifying the solid electrolyte interphase (SEI) to address issues arising from silicon’s significant volume expansion. It promotes a transition from static, stabilizing strategies to dynamic, adaptive interfaces that evolve in accordance with silicon’s behavior during cycling. The discussion highlights key advancements in the dimensional evolution of SEI layers, transitioning from brittle 3D structures to more stable, conductive 2D configurations. The significance of hierarchical chemical coupling, integrating rigid inorganic components such as LiF with flexible polymer matrices, is highlighted for its role in ensuring mechanical stability and ionic conductivity. The integration of self-healing strategies and dynamic chemical bonds and the system-level integration of electrolytes, electrodes, and interfaces are critical for developing intelligent silicon anodes capable of adapting to mechanical and electrochemical stresses. This novel approach has the potential to enhance the performance and longevity of silicon-based anodes in next-generation batteries.

## Public summary

• The evolution of silicon anodes: adaptive interfaces provide a dynamic solution to severe volume expansion.
• Dimensional regulation of SEI: transitioning from 3D porous networks to 2D compact configurations.
• Hierarchical coupling of rigid inorganics and flexible polymers benefits interfacial stability.
• System-level integration, self-healing strategies, and data-driven design advance silicon anodes.

## Graphical abstract

**Figure:**
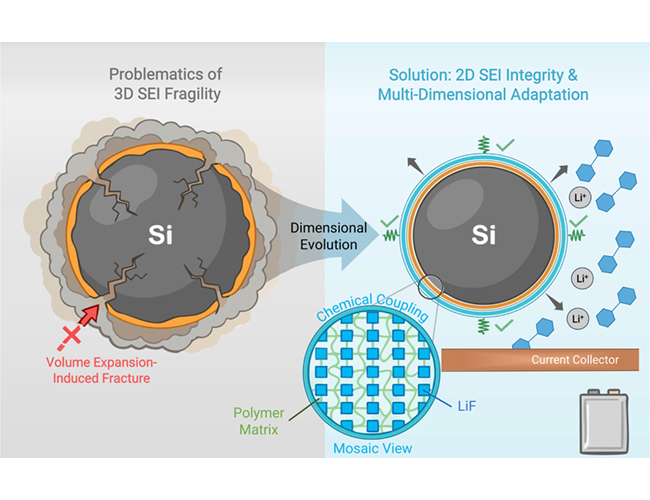

## Introduction

Silicon stands out as a leading anode candidate for next-generation lithium-ion batteries (LIBs), attributed to its ultrahigh theoretical capacity (4,200 mAh g^−1^), low lithiation potential (∼0.4 V vs. Li^+^/Li), and abundant reserves.^1^ This exceptional capacity far exceeds that of conventional graphite anodes (372 mAh g^−1^), making silicon indispensable for surpassing the energy density limit of current commercial LIBs.^2^ However, its practical deployment remains challenging after two decades of intensive research, primarily due to the drastic volumetric expansion (up to 400%) during lithiation-delithiation cycles, as shown in Table 1.^3^^,^^4^^,^^5^ This cyclic expansion-contraction pulverizes the silicon matrix, disrupts electrical pathways, and repeatedly fractures the solid electrolyte interphase (SEI). Every fracture exposes new surfaces that continuously interact with the electrolyte, depleting both lithium and solvent, leading to erratic Coulombic efficiency, excessive SEI buildup, and accelerated capacity degradation.^6^^,^^7^

**Table 1:** Comparison of theoretical capacities and volume expansions among Li-Si phases

Lithiation phase	Crystal ystem (space group)	Density (g cm^−3^)	Theoretical specific capacity (mAh g^−1^)	Corresponding volume expansion (%)
Si	cubic (*Fd*$\bar{3}$*m*)	2.33	0	0
Li_12_Si_7_	orthorhombic (*Pnma*)	1.53	1,635	160
Li_7_Si_3_	trigonal (P3_2_12)	1.46	2,320	222
Li_13_Si_4_	orthorhombic (*Pbam*)	1.25	3,100	389
Li_15_Si_4_	cubic (*I*$\bar{4}$3*d*)	1.19	3,579	280–300
Li_22_Si_5_	cubic (*F*$\bar{4}$3*m*)	1.18	4,200	400

During the early 2010s, mechanical mitigation strategies dominated the field,^8^^,^^9^ including nanostructuring and carbon coating.^10^^,^^11^^,^^12^^,^^13^ Despite these methods demonstrating remarkable early-cycle stability in laboratory cells, their scalability and volumetric energy density were constrained by the abundance of inactive components and low tap density. More critically, these strategies failed to address the SEI’s intrinsic dynamic instability, which constitutes the fundamental bottleneck to silicon’s practical deployment.^14^^,^^15^ This critical observation has shifted the research focus from structural engineering to interfacial regulation: the SEI, previously regarded as an unavoidable byproduct of electrolyte decomposition, is now recognized as a key design element that must co-evolve with the silicon substrate. This conceptual shift, from passive stabilization to active adaptation, represents a pivotal advancement in addressing silicon’s intrinsic challenges.

Recent progress in electrolyte and interphase engineering has facilitated this transformative shift. In highly fluorinated or concentrated electrolytes, solvent reduction is inhibited, leading to the predominance of anion-derived species (e.g., LiF, Li_2_O, and Li_3_N) in SEI formation, resulting in thin, dense, and ionically conductive interphases.^14^^,^^16^ Similarly, polymer-based or quasi-solid electrolytes confine reactions to the electrode surface, yielding conformal, flexible 2D SEI layers, in sharp contrast to the thick, porous 3D films formed in traditional carbonate systems.^17^^,^^18^ Collectively, these advances demonstrate the feasibility of engineering adaptive SEI layers that preserve structural integrity and ionic transport channels amid severe volumetric fluctuations. The emerging concept of interface adaptation encompasses three interrelated dimensions: (1) chemical adaptivity, characterized by dynamic redox or self-repairing reactions that sustain the composition and stability of the SEI; (2) mechanical adaptivity, involving hierarchical modulus gradients or elastic polymer frameworks that mitigate strain; and (3) system adaptivity, realized through the co-design of electrolytes and electrodes to modulate interfacial reactions under operational cell conditions.

**Figure 1:**
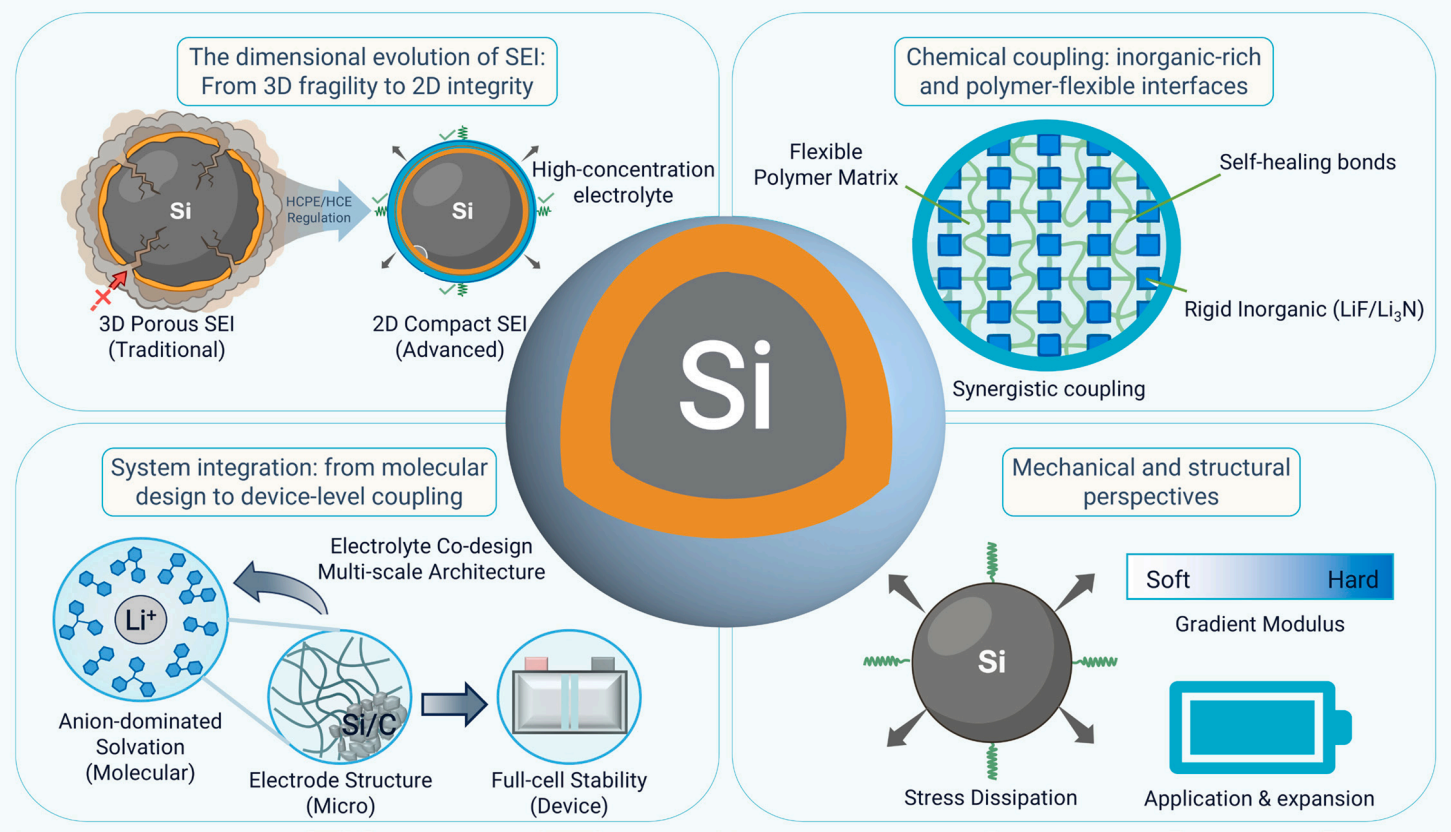
The unified design framework for interface-adaptive silicon anodes

This review outlines the conceptual development and proposes a unified framework for the design and understanding of interface-adaptive silicon anodes. We first analyze the electrochemical and mechanical implications of SEI dimensional control, transitioning from brittle 3D layers to compact 2D configurations. We then explore chemical coupling strategies that integrate inorganic rigidity with polymeric elasticity within the SEI. Next, we evaluate the significance of system-level integration, encompassing electrolyte selection, solid-liquid coupling, and electrode architecture. Emerging trends in self-healing and data-driven interfacial design are also emphasized. The overarching goal is to establish a design philosophy enabling the transformation of silicon from a fragile, high-capacity material into a resilient, adaptive, and intelligent anode system for next-generation high-energy batteries (Figure 1).

## The dimensional evolution of SEI: From 3D fragility to 2D integrity

**Figure 2:**
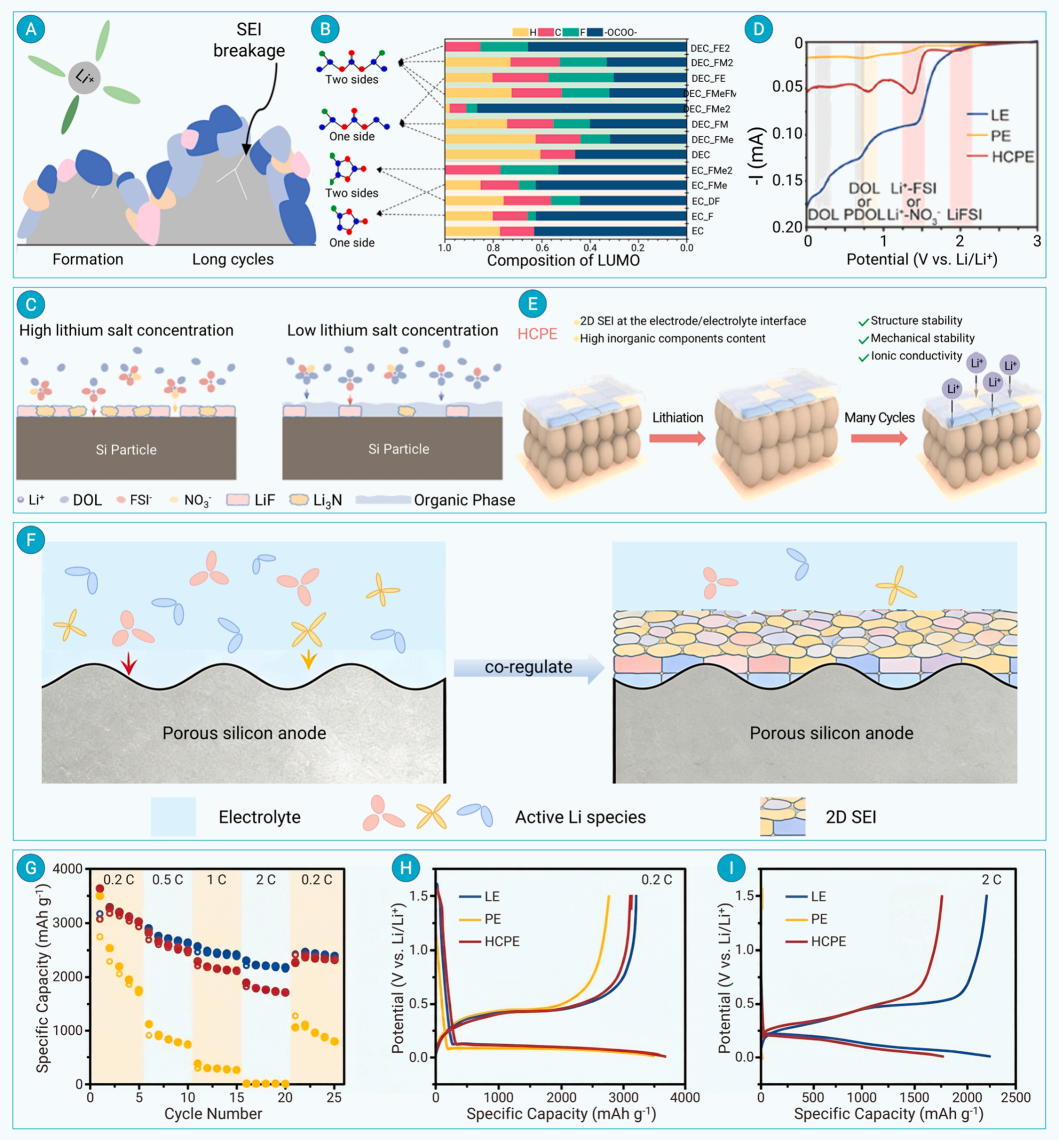
Dimensional evolution of SEI configurations driven by substrate geometry and interfacial kinetics (A) Schematic of SEI breakage.^21^ (B) Orbital contributions of LUMO.^22^ (C) Evolution of SEI components. (D) LSV curves for different electrolytes. (E) Evolution of SEI in HCPE. (F) Schematic illustration of the synergistic regulation of SEI dimensionality by substrate geometry and interfacial reaction kinetics. (G–I) Rate performance (G), charge and discharge curves at 0.2 C (H), and charge and discharge curves at 2 C (I) for cells with different electrolytes.^27.^

The idea of dimensional control in SEI design is among the most revolutionary advancements in silicon anode technology. Conventional SEI layers are usually 3D porous networks made of both organic and inorganic breakdown products in carbonate-based electrolytes. These thick and heterogeneous layers are intrinsically unstable due to their uneven morphology and weak bonding network, which makes them incapable of withstanding the repetitive expansion and contraction of silicon during lithiation-delithiation.^19^^,^^20^ When the SEI cracks, new silicon surfaces are exposed (Figure 2A).^21^ These surfaces then react further with the electrolyte, producing additional breakdown products and continuing the “crack-repair-crack” cycle. As a result, 3D SEI coatings thicken irreversibly, increasing resistance and ultimately blocking ion transport routes, leading to a sharp drop in capacity. From a thermodynamic point of view, this instability results from unconstrained electrolyte reduction kinetics. In liquid carbonate systems, as shown in Figure 2B, solvent molecules with low lowest unoccupied molecular orbital (LUMO) energies can be effectively decreased at potentials lower than 1.0 V vs. Li^+^/Li.^22^ On the dynamically changing Si surface, the breakdown products precipitate heterogeneously, forming an uneven, nonuniform layer with both electrically insulating and ionically resistive areas. These SEI layers act as partial barriers rather than selective membranes, limiting Li^+^ mobility and potentially causing significant hysteresis.

Recent advancements have reshaped the field by introducing highly concentrated electrolytes (HCEs) and their polymer-based variants, high-concentration polymer electrolytes (HCPEs), which together drive the dimensional evolution of SEI from brittle 3D to robust 2D architectures. Typical HCE systems lay the groundwork for 2D SEI formation. For instance, LiFSI-propylene carbonate with a salt-to-solvent molar ratio of 1:2 restructures Li^+^ solvation to foster anion-dominated reduction,^23^ while cyclic tetrahydrofuran-based HCEs leverage weak solvating power and strong polymerization to induce thin LiF-rich inorganic-polymeric interphases.^24^^,^^25^ HCPEs with a salt-to-solvent ratio exceeding 1:2 further refine and elevate this dimensional control, emerging as the premier strategy for reliable 2D SEI fabrication through synergistic integration of solvation engineering, polymerization dynamics regulation, and matrix confinement effects.^26^ A representative HCPE design incorporates 6 M LiFSI with 1,3-dioxolane as the polymer precursor, where NO_3_^−^ anions play a pivotal regulatory role by retarding 1,3-dioxolane polymerization kinetics to enable high salt dissolution and uniform solvation distribution.^27^ A high LiFSI concentration reconfigures the solvation environment by competing with 1,3-dioxolane for Li^+^ coordination, alleviating 1,3-dioxolane and poly-1,3-dioxolane decomposition while directing preferential reduction of FSI^−^ anions.^28^^,^^29^ This selective anion reduction induces the formation of LiF and Li_3_N-rich nanodomains that act as mechanical reinforcement within the SEI, while the polymer matrix generated via controlled 1,3-dioxolane polymerization provides elastic buffering.^30^ The inherent high viscosity and restricted diffusion of the HCPE matrix further confine electrolyte decomposition to a localized interfacial region, suppressing bulk side reactions and ensuring the SEI remains compact, conformal, and free of porosity.^31^ Unlike HCEs that rely primarily on solvation restructuring, HCPEs achieve precise control over SEI dimensionality through multi-factor synergy: solvation environment dictates the reduction pathway, polymerization dynamics modulate SEI uniformity, and polymer matrix confinement eliminates 3D porous formation. Together, these features enable HCPE-derived 2D SEI to not only resist cracking during Si volume expansion but also sustain continuous Li^+^ transport channels, addressing the fundamental limitations of 3D SEI and underscoring HCPE as the superior approach for interface dimensional control in Si anodes.

The transition from 3D to 2D SEI significantly alters interfacial behavior.^27^^,^^32^ 2D SEI layers measuring 5–10 nm in thickness exhibit dense LiF and Li_2_O nanodomains within flexible polymer matrices, which integrate high modulus, ionic conductivity, and chemical stability as shown in Figures 2C and 2D.^27^ Their compact morphology inhibits electrolyte infiltration and parasitic reactions, thereby preserving conformity after extended cycling, as evidenced by cryo-transmission electron microscopy (cryo-TEM) and *in situ* tomography. Li^+^ migration in 2D films occurs along grain boundaries and polymeric channels, characterized by reduced activation barriers of less than 0.3 eV, which facilitates rapid kinetics under compressive stress. Finite-element analysis indicates that thinner SEI layers exhibit reduced stress localization and lower strain gradients, while thicker 3D films tend to accumulate tensile stress and experience delamination. The controlled *in situ* polymerization of dioxolane (DOL)-based electrolytes illustrates this principle. LiNO_3_ or LiFSI additives influence DOL polymerization and promote the development of LiF/Li_3_N-rich 2D interphases, resulting in chemically dense and mechanically flexible layers. LiF-dominant 2D structures also appear in ionic liquid and fluorinated carbonate electrolytes, as strong electron-withdrawing solvents promote reduction, leading to anion decomposition. Eliminating porosity and optimizing composition in 2D SEI architectures leads to improved mechanical resilience and Li^+^ transport, highlighting dimensional control as a critical strategy for stabilizing silicon-electrolyte interfaces, as shown in Figure 2E.^27^ Consequently, the selection among these advanced systems is dictated by distinct application requirements. HCPEs, yielding elastic 2D SEI, are particularly suited for high-loading electrodes where long-term mechanical stability is paramount. Fluorinated carbonate electrolytes, forming thin and highly ion-conductive interphases, excel in applications demanding high-rate capability. Ionic liquids, with their inherent chemical and thermal stability, offer unique advantages for operation under harsh conditions, such as elevated temperatures or high voltages.

Surface topology gives an additional dimension of control.^33^ The quasi-2D formation mode is efficiently facilitated by prepatterned or nanoporous silicon structures, which enhance uniform SEI development and lateral stress distribution. For instance, silicon nanopillar arrays fabricated via nanoimprint lithography combined with reactive-ion etching leverage well-defined surface geometries to disperse interfacial stress, while carbon-coated mesoporous silicon prepared through an amine-assisted solution reaction of Mg_2_Si with HSiCl_3_ and porous silicon prepared by NaCl-assisted magnesiothermic reduction rely on tailored pore structures to promote homogeneous SEI growth.^34^^,^^35^^,^^36^ These examples demonstrate that topological design offers a physical pathway to direct SEI growth, complementing the chemical pathway governed by electrolyte engineering discussed earlier. The superior electrochemical performance of these structurally engineered materials, such as high reversible capacities, stable cycling retention, and excellent rate capabilities, directly verifies that this physical pathway modulates SEI dimensionality by optimizing reaction localization and stress distribution. Together with the previously discussed electrolyte-driven control of interfacial kinetics, these findings underscore that the SEI’s dimensionality is not an intrinsic material property but a designable feature governed collectively by substrate architecture and interfacial reaction kinetics.^37^ This dimensional control is realized through two synergistic mechanisms. Substrate topology provides a geometric template that homogenizes interfacial stress and guides uniform SEI nucleation. Concurrently, interfacial reaction kinetics, which are dictated by electrolyte composition, control the chemical pathway of SEI formation, thereby determining its ultimate compactness and composition. Key kinetic parameters encompass solvation structure, polymerization dynamics, and reaction localization. The deliberate engineering of these pathways through advanced electrolyte systems is therefore fundamental to suppressing 3D growth and enabling the formation of compact 2D interphases.^38^ Figure 2F schematically illustrates the synergistic regulation of SEI dimensionality by substrate geometry and interfacial reaction kinetics. The interplay between these physical and chemical levers, in which substrate geometry dictates where and how stress is managed while interfacial kinetics determines what chemically forms, is precisely what enables the rational design of SEI dimensionality.

The shift to 2D SEI represents a pivotal moment for silicon anodes in practical applications. It ensures constant cycling at elevated areal capacities (>4 mAh cm^−2^) and low N/P ratios, crucial for commercial viability, by minimizing interfacial heterogeneity (Figures 2G–2I).^27^ Crucially, the principle of dimensional regulation provides a cohesive design guideline for future interphase engineering: stability is achieved through adaptability instead of thickness. The next generation of silicon interfaces should be thin, dynamic, and self-regulating rather than relying on thicker passivation coatings.^39^ This insight indicates that the optimal SEI is not a static barrier but a dynamic membrane that co-evolves with the active material, thus integrating surface chemistry with mechanical design. In the following section, we expand this concept from dimensional to chemical coupling, where the synergistic combination of inorganic and polymeric components provides enhanced interfacial flexibility.

## Chemical coupling: Inorganic-rich and polymer-flexible interfaces

The SEI’s mechanical and electrochemical stability is contingent upon the chemical coupling among its constituent phases, in addition to its dimensional compactness. A single-component SEI, regardless of whether it is entirely inorganic or entirely polymeric, is incapable of simultaneously providing high rigidity, elasticity, and ionic conductivity. Consequently, chemically integrated hybrid interphases have emerged as a universal approach to achieving long-term stability in silicon anodes.^40^ Their functionality can be succinctly described as three critical components.

### Inorganic backbone for passivation and rigidity

**Figure 3:**
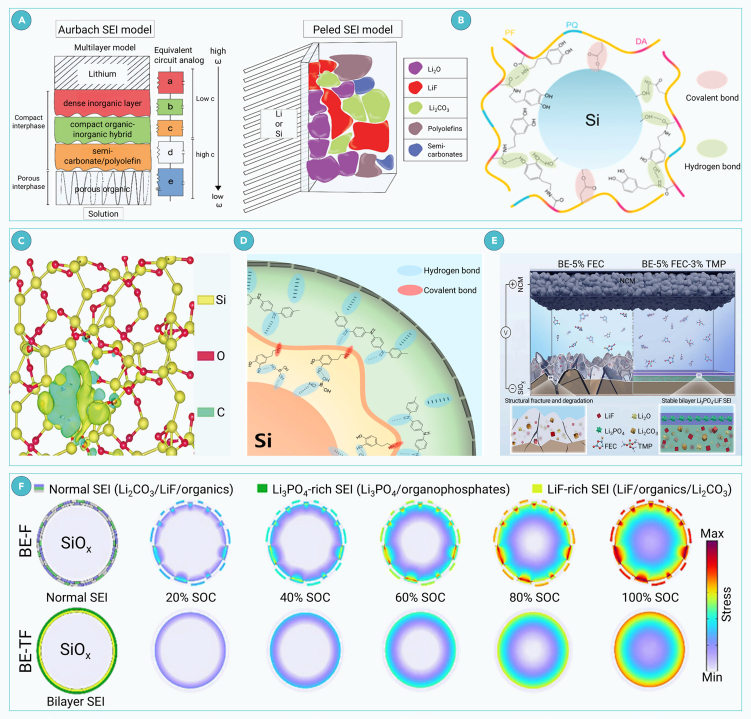
Hierarchical chemical coupling of inorganic domains and flexible polymer matrices for interfacial stability (A) Schematic of the multilayer model for lithium electrodes (Aurbach model) and the “mosaic” disordered poly-heterogeneous microphase SEI model for lithium or carbon electrodes (Peled model).^41^ (B) Scheme of multiple networks constructed within Si/PFPQDA electrodes, PFPQDA denotes dopamine-functionalized fluorene structure units introduced into a polyfluorene-typed copolymer.^45^ (C) Radial dispersion function *g*_Li–Si/__O_.^47^ (D) Proposed ideal polymer coating system for the Si anode in LIB.^51^ (E) Schematic of a full-cell based on NCM||micron-sized SiO_*x*_. (F) Finite-element simulation of stress evolution in SiO_*x*_ anodes with BE-F (base carbonate electrolyte with 5 wt% FEC) and BE-TF (base carbonate electrolyte with 3 wt% trimethyl phosphate and 5 wt% FEC) electrolytes.^52.^

The structural skeleton of the SEI is composed of inorganic components, including LiF, Li_2_O, Li_3_N, and Li_3_P, as substantiated by the classic models in Figure 3A, where Aurbach’s multilayer model identifies the innermost layer as the dense inorganic phase adjacent to the electrode, while Peled’s mosaic model features discrete inorganic domains forming the structural core of the disordered SEI.^41^ They effectively suppress parasitic electrolyte decomposition and electron leakage by providing high modulus, superior chemical inertness, and electronic insulation. Among these, LiF is critical because it is a dense, chemically stable layer that resists continuous disintegration.^42^ Nevertheless, brittleness and inadequate Li^+^ transport are the consequences of an overabundance of LiF. Therefore, optimal interphases are composed of nanocrystalline LiF clusters (2–5 nm) embedded in gentler matrices, thereby achieving a balance between rigidity and ion conduction.

### Polymeric matrix for adhesion and elasticity

Flexible polymeric matrices fulfill two indispensable roles in silicon anodes: intrinsic mechanical buffering and interfacial adhesion. The buffering capability originates from the polymer’s intrinsic structural design. High chain segment mobility, provided by backbones containing C–O–C linkages (e.g., in polyethers such as poly (DOL)) or Si–O–Si linkages (e.g., in polysiloxanes), enables reversible deformation to dissipate stress.^43^^,^^44^ A moderate crosslinking density is concurrently essential to balance this elasticity with structural integrity. Furthermore, ionic conduction within this flexible network is facilitated by the oxygen atoms in these linkages acting as coordination sites for Li^+^ ions. The polymer’s backbone structure and moderate crosslinking network, which enable reversible deformation and balanced integrity, are schematically illustrated in Figure 3B.^45^ The spatial correlation between Li^+^ ions and the polymer’s functional groups, directly reflected by the radial distribution function *g*_Li–Si/O_ in Figure 3C, confirms this interaction at the atomic level. This evidence underpins the polymer’s critical ability to support Li^+^ transport while retaining its flexible structure, reconciling mechanical and ionic conductive functions.^46^^,^^47^

The second role, robust interfacial adhesion, is achieved through deliberate chemical design of the polymer to bond with silicon active particles. This involves incorporating specific functional groups capable of targeted interactions with the particle surface.^48^^,^^49^ Polymers bearing silanol or aminosilane groups can form robust covalent Si–O–Si or Si–N bonds via condensation reactions with surface hydroxyl groups, providing strong chemical anchoring. Adhesion is further reinforced by non-covalent interactions, such as hydrogen bonding and π–π stacking, from polar segments or aromatic moieties within polymers.^50^ The hierarchical binding networks formed by these covalent and non-covalent interactions are visualized in Figure 3D.^51^ The integration of these tailored chemical features with the aforementioned structural design ensures intimate polymer-particle contact. This suppresses void formation, maintains continuous electrical and ionic pathways, and mitigates electrolyte infiltration, thereby ensuring long-term cycling stability.

### Self-adaptivity and hierarchical chemical coupling

Bilayer LiF–Si–O–Si architectures (a LiF-rich inorganic layer coupled with a polymer matrix featuring Si–O–Si covalent linkages) represent some of the most durable SEIs. This design integrates the mechanical strength of the inorganic phase with the flexibility of the polymer phase through hierarchical chemical coupling. The inorganic phase, mainly LiF, contributes to high modulus and structural integrity, whereas the polymeric phase regulates the dynamics of Li^+^ solvation and influences interfacial adhesion energy. The “inner-hard/outer-soft” configurations maintain interfacial integrity throughout the anode’s repeated volume expansion and contraction (Figure 3E).^52^ Figure 3F confirms this mechanical advantage via finite-element simulation: the bilayer Li_3_PO_4_-LiF SEI from the BE-TF electrolyte achieves uniform stress distribution, while the normal SEI from the BE-F electrolyte shows stress concentration and fracture, supporting hierarchical chemical coupling’s role in adaptive stability. Recent trends in chemically coupled SEI design further integrate dynamic chemical bonds, including imine hydrogen and disulfide linkages, to enhance self-healing capability and reinforce the interphase’s adaptive response to mechanical stress.^53^^,^^54^ These dynamic bonds exhibit distinct stability during long-term cycling, directly governing interfacial integrity. Hydrogen bonds in polymer matrices undergo continuous breaking and reforming in response to silicon volume fluctuations, facilitating stress dissipation.^55^ Prolonged electrolyte exposure weakens their bonding efficiency, gradually reducing the self-healing capacity over extended cycling. In contrast, disulfide bonds maintain reversible cleavage and reformation due to their inherent resistance to electrolyte erosion, thereby sustaining interfacial cohesion. Imine bonds enable efficient early-cycle crack repair but tend to undergo irreversible hydrolysis in the presence of trace moisture in electrolytes, losing their dynamic reversibility.^56^ The evolution of these dynamic bonds determines the interphase’s long-term adaptability. Robust bonds sustain repeated structural adjustments to preserve interface integrity, while labile ones require synergistic stabilization with inorganic domains to maintain cohesion.

In essence, the chemical coupling strategy converts the SEI into a multifunctional, active interface that coordinates ionic transport, stress distribution, and chemical reactivity. This composite paradigm establishes the groundwork for the subsequent phase of interfacial design, system-level integration. In this phase, the electrolyte composition, electrode structure, and interphase chemistry are co-optimized to guarantee full-cell stability and scalability.

## System integration: From molecular design to device-level coupling

Transitioning silicon anodes from conceptual materials to industrially viable systems is contingent upon system-level integration. To accommodate the dynamic electro-chemo-mechanical behavior of silicon, the interface, electrolyte, and electrode architecture must function in a synergistic manner. These three fundamental design axes can be used to rationalize this integration.

### Electrolyte-interphase co-design

Fundamentally, the solvation environment of Li^+^ determines the interfacial chemistry of silicon, and electrolyte-interphase co-design hinges on a core guiding principle: synergistic tuning of solvation structure and interfacial reaction localization to match the dynamic electro-chemo-mechanical behavior of silicon. Conventional carbonate electrolytes produce unstable, organic-rich SEI films due to unrestrained solvent reduction, as their low LUMO energy solvents undergo indiscriminate decomposition across both silicon surfaces and bulk electrolyte.^57^ In contrast, anion-dominated solvation structures derived from high-concentration or fluorinated ether electrolytes redirect the reduction pathway toward salt anions such as FSI^−^ and DFOB^–^, yielding dense, LiF-enriched SEI layers that resist parasitic reactions.

**Figure 4:**
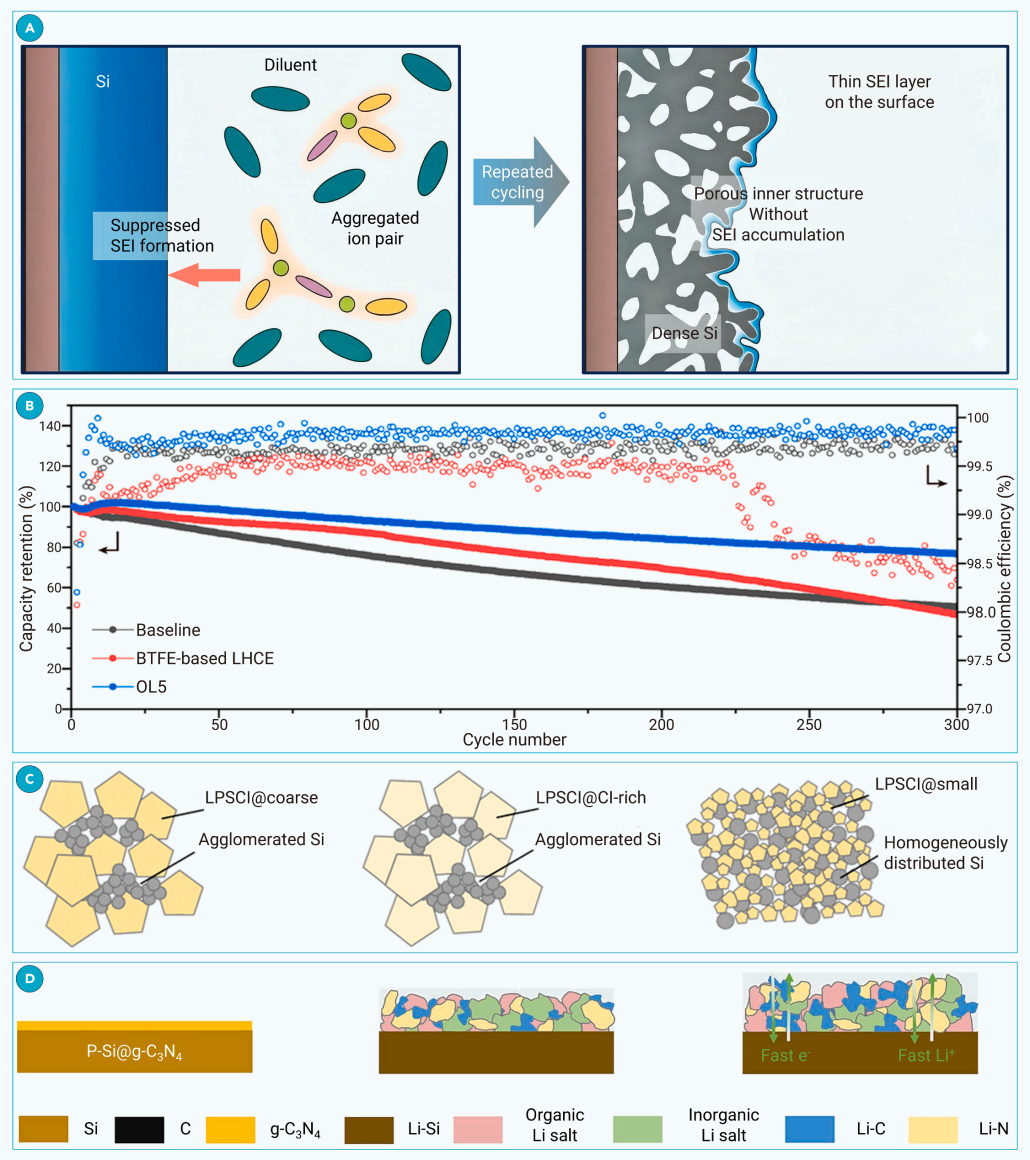
System-level integration of localized electrolytes and composite architectures for stable silicon anodes (A) Effect of baseline and 1H,1H,5H-octafluoropentyl 1,1,2,2-tetrafluoroethyl ether-based LHCE electrolytes on Si anode degradation. (B) Long-term cycling performance and CE of Si/graphite||NMC532 full cells with different electrolytes at 45°C.^61^ (C) Schematic of microstructures in various Si/LPSCl composites.^66^ (D) Schematic diagram of SEI composition for p-Si@g-C_3_N_4_, showing organic/inorganic lithium salts formed by electrolyte decomposition and Li-C and Li-N formed by embedding lithium in g-C_3_N_4_.^76.^

Localized high-concentration electrolytes (LHCEs) exemplify this co-design principle through tailored component reactivity. For instance, a dual-salt ether-based LHCE incorporating fluoroethylene carbonate (FEC; termed D-LHCE-F) modulates the reduction kinetics of electrolyte components: FEC interacts synergistically with the dual-salt solvation shell to promote selective formation of LiF-rich SEI, while the ether-based solvent framework enhances the SEI’s mechanical flexibility by reducing crosslinking density. This synergy enables the interphase to accommodate silicon’s substantial volume variation without structural failure.^58^ Conversely, an FEC-free LHCE eliminates competitive reduction between additive and salt anions, facilitating the formation of uniform, anion-derived interphase layers on both cathode and anode that block electrode crosstalk and suppress interfacial impedance growth during storage at 55°C.^59^ These LHCE systems balance minimal gas evolution and high Coulombic efficiency, as illustrated in Figures 4A and 4B.^60^^,^^61^ Polymer and gel electrolytes extend this co-design strategy through interfacial reaction localization and solvent confinement, establishing a clear causal link between reaction zone restriction and composite interphase formation. Their high viscosity and crosslinked network structures immobilize solvent molecules, limiting electrolyte component diffusion and confining reduction reactions to the immediate vicinity of the silicon surface. This suppresses bulk-phase decomposition, which would otherwise produce porous heterogeneous SEI. This localized reaction environment forces salt anions, such as FSI^−^ and NO_3_^−^, to dominate the reduction process, as solvent molecules are no longer freely available for unrestrained decomposition. Simultaneous polymer chains such as poly (DOL) and poly (siloxane) or gel networks interact with Li^+^ ions to reshape their solvation shells, promoting co-deposition of Li_3_N and LiF as discrete nanodomains within the polymer matrix.^62^^,^^63^ The polymer fraction further modulates SEI mechanical properties. Flexible polymer backbones reduce interfacial strain during silicon expansion, while inorganic Li_3_N/LiF domains provide structural rigidity, yielding a composite interphase integrating chemical stability and mechanical adaptability.^64^ This mechanism underscores the co-design logic for polymer/gel electrolytes. Solvent confinement ensures anion-dominated reduction, the basis for Li_3_N/LiF formation, while polymer-silicon surface interactions tailor SEI structure to withstand dynamic volume changes.

### Coupling of solid-state and hybrid systems

As liquid systems approach their intrinsic limits, solid-state and quasi-solid electrolytes offer novel paradigms for mechanical and chemical stability. Gel polymer electrolytes and polymer-ceramic composites are capable of sustaining continuous ionic conduction while accommodating the volumetric expansion of Si.^65^ Soft polymer matrices could absorb mechanical strain, while rigid inorganic fillers (e.g., Li_7_La_3_Zr_e_O_12_ and Li_1.3_Al_0.3_Ti_1.7_(PO_e_)_3_) provide rapid ion channels. Sulfide-based solid-state systems (Li_6_PS_5_Cl and Li_10_GeP_2_S_12_) naturally form Li_2_S/Li_3_P/LiF self-limiting passivation layers that stabilize the Si interface during cycling, as illustrated in Figure 4C.^66^ Within this architecture, the homogeneous distribution of Si particles ensures intimate contact with the sulfide electrolyte matrix. Such microstructural continuity is instrumental, as it facilitates the uniform formation of self-limiting interphases while mechanically mitigating crack propagation, thereby underpinning the enhanced interfacial stability characteristic of these systems.^67^ Nevertheless, expansion of large Si may result in delamination due to high modulus values (10–20 GPa).^68^ By incorporating buffer interlayers, such as elastic polymers, 2D nanosheets, or Li_3_N films, mechanical stress absorbers are maintained, ensuring ionic continuity and contact. Elastic polymers such as polyether and polysiloxane function primarily as compliant stress dissipaters, absorbing strain through the reversible stretching of molecular networks enabled by dynamic bonds, and their low shear modulus (below 10 MPa) allows them to conform to volume changes and maintain essential physical contact for ion conduction.^69^ Solid-state silicon systems can coexist with mechanical and ionic compatibility, as demonstrated by this “soft-hard coupling” strategy.^70^ In contrast, 2D nanosheets, such as graphene and MXene, act as integrity-preserving strain buffers, alleviating stress via interlayer sliding, while their high in-plane stiffness (exceeding 100 GPa) mechanically supports the electrode to sustain ion-percolation pathways.^71^^,^^72^ The Li_3_N film-like materials serve a dual role as conductive buffers, providing intrinsic ionic conductivity (around 10^−1^ S·cm^−1^) coupled with plastic deformability to relieve stress without interrupting their own ion-transport function.^73^^,^^74^^,^^75^ Figure 4D schematically verifies this cooperative design, showing how a g-C_3_N_4_-derived hybrid SEI mimics these principles to mitigate interfacial stress concentration.^76^ Therefore, by leveraging mechanisms from polymeric chain dynamics to lattice flexibility, these interlayers exemplify the soft-hard coupling strategy, validating that decoupling mechanical and ionic transport demands is fundamental to stabilizing solid-state silicon interfaces.

### Practical metrics and multiscale structural integration

To achieve effective system integration, it is necessary to coordinate all components within the electrode and the cell, as well as local chemistry. Practical performance is determined by a multiscale architecture that encompasses nanoscale SEI regulation, microscale conductive frameworks, and cell-level stack design.^77^ This integrated approach is evidenced by concurrent advances in interfacial and structural engineering. One strategy focuses on constructing a robust artificial SEI via a conductive polymer layer, yielding a uniform LiF-rich interface to ensure ionic transport stability.^78^^,^^79^ Similarly, modifying silicon particle surfaces with a sulfur-doped carbon layer (SDCL) promotes the formation of a stable SEI while enhancing electronic conductivity and reducing lithium-ion diffusion barriers via modulated electron cloud distribution.^80^ Another complementary strategy employs a hierarchical design that confines porous silicon within a 3D conductive network of graphene oxide and carbon nanotubes, thereby addressing volume buffering and electron conduction in tandem.^81^ At the microscale, stress distribution can be uniformized by porous carbon, MXene, or graphene scaffolds.^82^ The integration of N-doped porous vertical graphene sheets into silicon-graphite anodes provides a pertinent example, where tailored porosity and directional channels efficiently buffer mechanical stress under practical conditions.^83^ Likewise, embedding silicon nanoparticles into an expanded graphite/pitch-derived carbon (EGC) matrix leverages the tailored porous architecture and robust carbon framework of EGC to synergistically buffer the volume variation of silicon during cycling while facilitating ion diffusion and electron transport.^84^ Concurrently, elastic binders and graded silicon concentrations ensure mechanical integrity throughout the electrode thickness. Advanced binders based on interwoven polyacrylic acid and poly(urea-urethane) elastomer networks exemplify how molecular design translates to macroscopic elasticity, preserving electrode cohesion during cycling.^85^

Integration quality is ultimately assessed by practical metrics, which include a high areal capacity (>4 mAh cm^−2^), thin electrodes (<100 μm), a limited electrolyte ratio (E/C < 2 g Ah^−1^), and stable full-cell cycling (>500 cycles, >80% retention).^86^ Chemistry, mechanics, and architecture must be coordinated to achieve these benchmarks. Electrolyte-interface pair optimization is further expedited by the convergence of *operando* spectroscopy, machine learning, and experimental data, which signifies the transition of silicon anodes from material innovation to systemic design.^87^

## Mechanical and structural perspectives: Understanding failure and adaptation

Mechanical stability serves as the prerequisite for the long-term reliability of silicon anodes. Although SEI deterioration has been reduced by chemical and interfacial advancements, structural failure remains unavoidable if mechanical coupling across the electrode system is not properly engineered.^88^ Therefore, it is essential to have a thorough understanding of stress creation, propagation, and accommodation. Three related viewpoints can be used to summarize this difficulty.

### Stress evolution and mechanical mismatch

**Figure 5:**
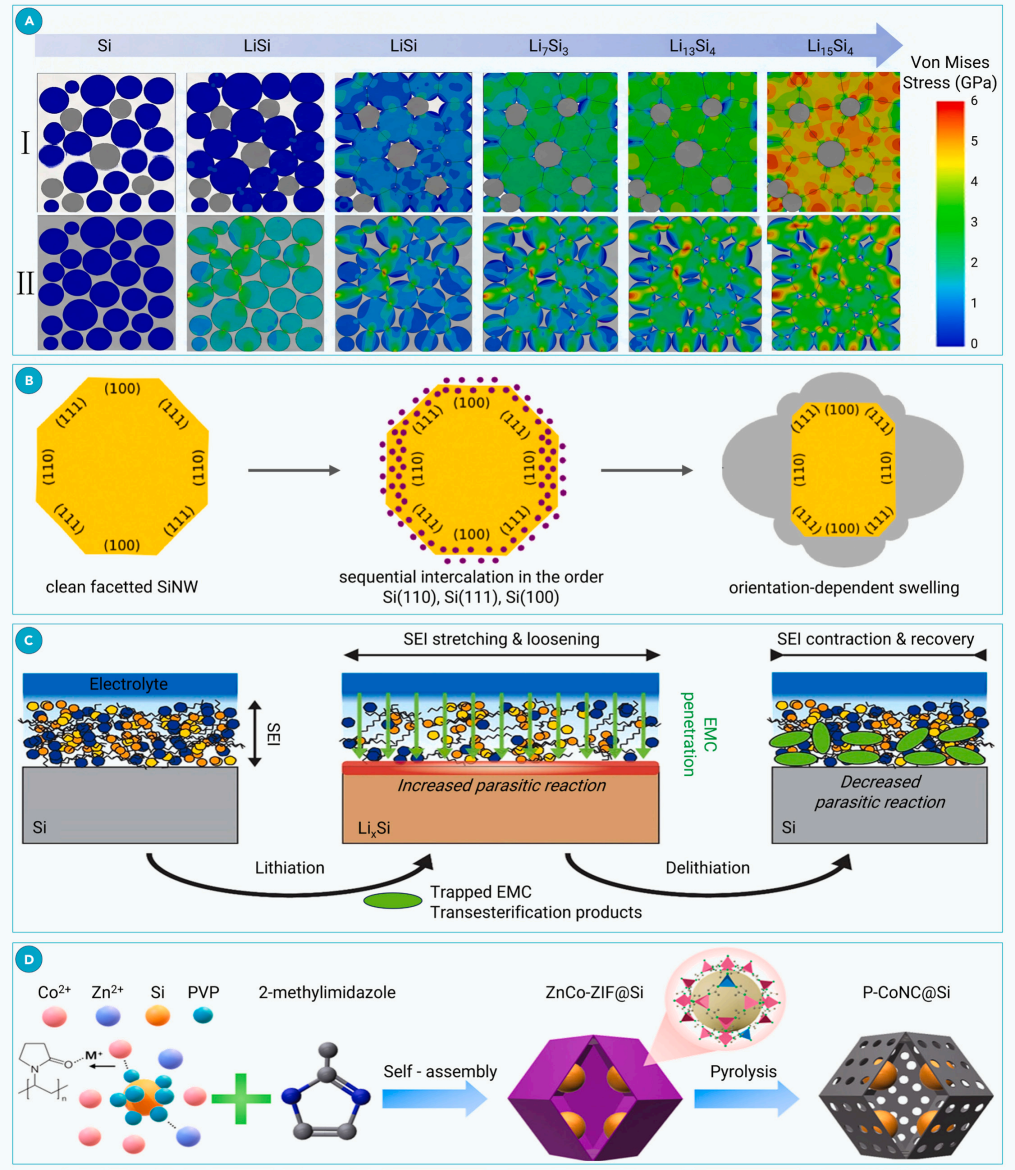
Multiscale mechanical failure mechanisms and corresponding structural adaptation strategies (A) Stress distribution and evolution in the μm-Si electrode with Li_6_PS_5_Cl solid-state electrolyte (I) and elastic electrolyte (II), simulated using the finite-element method.^91^ (B) Atomistic view of the structural evolution of a faceted Si nanowire during lithiation.^96^ (C) SEI dynamic “breath” effect.^99^ (D) Schematic representation of the synthesis of hollow porous p-CoNC@Si.^104.^

Silicon undergoes a volumetric expansion of over 300% during complete lithiation, leading to internal stress gradients exceeding several gigapascals. Conventional designs exhibit a modulus mismatch between the rigid SEI, with a modulus of tens of GPa, and the expanding silicon core, with a modulus of approximately 10 GPa.^89^ This discrepancy results in tensile cracking and delamination. *Operando* optical and electron microscopy confirm that stresses accumulate cyclically, even in nanosized particles, leading to pulverization and SEI rupture. Finite-element analysis indicates that stress localization is most pronounced at particle necks and SEI-substrate interfaces, as shown in Figure 5A.^90^^,^^91^ Thicker 3D SEI layers enhance strain gradients, while thinner 2D layers with a graded modulus facilitate more uniform stress distribution. The mechanical mismatch delineates the transition from reversible to irreversible deformation, underscoring the need for elastic, compliant, and adhesive interfaces rather than solely strong or rigid ones.

To quantitatively evaluate such mechanical mismatch and guide interface design, interfacial adhesion energy and the modulus gradient emerge as critical parameters. Interfacial adhesion energy, which quantifies the bonding strength between the SEI and the silicon substrate, can be probed using techniques such as 180° peel tests and atomic force microscopy-based force spectroscopy.^92^ For engineered polymer-Si interfaces, effective interfacial adhesion strength on the order of 0.5–2.5 N cm^−1^ can be achieved through optimal design. Concurrently, the modulus gradient across the SEI thickness is paramount for efficient stress dissipation. An exemplary design features a transition from a compliant outer polymeric layer (∼0.2–0.5 GPa) to a rigid inner LiF-rich layer. Notably, artificial gradient SEI layers incorporating such inorganic components can exhibit inner layer moduli of 6.5 GPa or higher.^93^^,^^94^ These quantifiable parameters establish a concrete framework for assessing mechanical adaptivity. Adequate adhesion energy ensures intimate contact that resists delamination, while a rationally designed modulus gradient mitigates stress concentration, collectively transforming the interface from a static barrier into a dynamically resilient structure.

### Failure mechanisms across the electrode hierarchy

Mechanical degradation manifests in distinct ways at various structural levels. Anisotropic lithiation at the particle scale leads to nonuniform expansion, resulting in radial cracking and intergranular fracture (Figure 5B).^95^^,^^96^ The collective expansion of silicon domains at the electrode scale generates shear stress at the interfaces of the binder and current collector.^97^ Furthermore, repeated electrode “breathing” may lead to deformation or collapse of polymer separators, thereby increasing internal resistance and heightening the risk of short-circuit formation, as shown in Figure 5C.^98^^,^^99^

Recent studies indicate that the separator modulus (>1 GPa) significantly affects the uniformity of Li^+^ flux; soft separators exhibit local deformation under pressure, leading to the formation of hotspots in ionic current.^100^^,^^101^ The use of mechanically reinforced or thermally crosslinked separators addresses this instability.^102^ Dynamic binders featuring hydrogen-bonding or supramolecular crosslinks preserve interparticle cohesion and allow for reversible strain, thereby effectively postponing electrode fatigue. The multiscale insights indicate that the mechanical failure of silicon is a systemic response rather than a localized event, involving all interfacial junctions.

### Structural adaptation and design strategies

The primary objective is to convert mechanical vulnerability into mechanical adaptability. Recent advances in electrode and SEI design are supported by this principle. Gradient-modulus SEI interface structures have a soft outer polymeric layer and a rigid inner LiF-rich layer for strain distribution and self-stress accommodation. Electrodes with yolk-shell Si@C particles, porous Si frameworks, and stretchable conductive networks (Carbon Nanotubes, graphene, and MXene) allow expansion while maintaining electrical continuity, as shown in Figure 5D.^103^^,^^104^ Elastic interlayers or 3D current collectors, such as copper foams and polymer-metal meshes, reduce macroscopic stress and electrode delamination at the system level. Combining density functional theory (DFT) with finite-element simulations enables predicted mechanical property adjustments, enabling the creation of materials for “modulus-matched” interfaces.^105^ Strategic stress direction and diffusion through hierarchical systems achieve mechanical flexibility. Changing from fighting deformation to promoting it is the mechanical equivalent of the adaptive interface. Chemical, structural, and mechanical methods produce a silicon anode with self-accommodation, long-term stability, and manufacturability for next-generation high-energy battery systems.

## Conclusion

The development of practical silicon anodes signifies not only a materials challenge but also a fundamental shift in our understanding of electrochemical interfaces. The concept of interface adaptation integrates chemical, mechanical, and system-level principles into a cohesive design philosophy. The convergence of these concepts, including dynamic SEI chemistry, multiscale mechanical orchestration, and data-guided integration, will enable silicon to surpass its historical limitations. The result will not be a “stable” anode in a static sense but rather an intelligent electrochemical system that exhibits self-regulation, resilience, and sustained functionality. The development of silicon anodes parallels the progression of biological systems; stability is achieved not through rigidity but through adaptability. Utilizing this principle represents a significant advancement in the development of sustainable, high-energy, and autonomous battery technologies.

## Outlook

**Figure 6:**
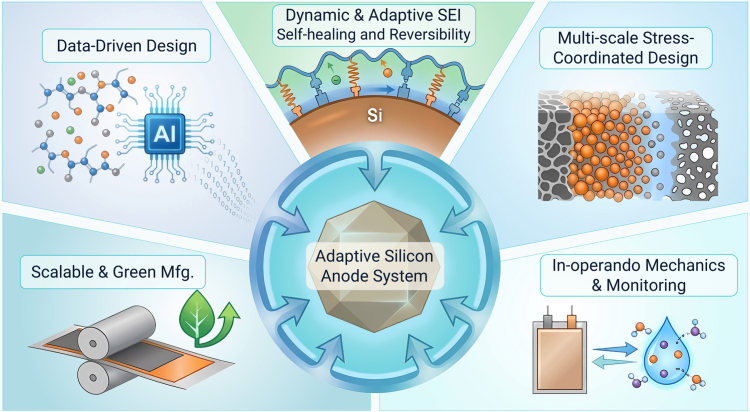
Toward intelligent and adaptive silicon anode systems A multi-dimensional synergistic design framework.

To realize commercial silicon anodes, we must move beyond passive stabilization and design intelligent, adaptive electrochemical systems. We propose that future research must synchronize efforts across five interrelated frontiers to resolve the intrinsic chemo-mechanical conflicts of silicon (Figure 6).

### Interfaces with intrinsic adaptive functionality

The SEI must be reimagined as a living membrane rather than a static passivation layer. Research should focus on incorporating dynamic covalent chemistry and supramolecular motifs that allow the interphase to autonomously self-heal and reconfigure its modulus in response to the volumetric breathing of silicon particles. Such molecular-level adaptivity is crucial for maintaining ionic homogeneity and mechanical robustness throughout battery cycling, even under varying thermal and electrochemical conditions.

### Scalable and sustainable manufacturing

Bridging the gap between coin cells and pouch cells necessitates manufacturing processes that are both scalable and environmentally benign. The field must prioritize the development of aqueous processing techniques and solvent-free electrode fabrication methods that eliminate the use of toxic organic solvents. Furthermore, the integration of bio-derived precursors and continuous pre-lithiation strategies into existing production lines will be critical to reducing the cost and carbon footprint of high-energy silicon anodes while ensuring strict quality control.

### Rational design via data-simulation fusion

The infinite compositional space of electrolytes and interphases demands a transition from empirical trial and error to predictive rational design. A closed-loop research paradigm that fuses high-throughput experimentation with machine learning and multiscale modeling is required to decipher complex structure-property relationships. This data-centric approach will enable the targeted engineering of solvation structures and interfacial chemistries that are precisely tuned to the dynamic needs of the silicon anode.

### *In operando* mechanics and dynamic regulation

A fundamental understanding of interfacial failure mechanisms hinges on the ability to probe mechanical and chemical evolution in real time. The development of *in operando* characterization techniques with high spatiotemporal resolution is emphasized to quantify dynamic stress distribution and modulus evolution during cycling. These diagnostic tools are indispensable for validating models and establishing feedback loops for active interfacial regulation.

### Stress-coordinated design in composite architectures

For prevailing silicon–carbon composite electrodes, structural integrity depends on the coordinated management of stress across multiple scales. Next-generation architectures should employ gradient designs and elastic buffering networks to delocalize strain, accommodating volume change without disrupting conductive pathways. The synergistic optimization of binders, conductive agents, and current collectors will be paramount to achieving high areal capacity and long-term cycling stability.

## Funding and acknowledgments

This work was financially supported by the 10.13039/501100001809National Natural Science Foundation of China (nos. 52271211 and 52171207) and the Science and Technology Innovation Program of Hunan Province (no. 2022RC3037), China.

## Author contributions

H.Y., Y.C., and Y.W. were responsible for the literature collection, writing of the initial draft, and figure/table preparation. S.M.T. revised the paper and supervised the research. H.Y., J.H., and Z.Y. supervised the overall structure and focus of the review, provided critical revisions, and were responsible for correspondence during submission and revision stages. All authors contributed to the manuscript and approved the final version.

## Declaration of interests

The authors declare no competing interests.
